# Unraveling Meso-Substituent Steric Effects on the Mechanism of Hydrogen Evolution Reaction in Ni^II^ Porphyrin Hydrides Using DFT Method

**DOI:** 10.3390/molecules29050986

**Published:** 2024-02-23

**Authors:** Xiaodong Li, Ailing Feng, Yanqing Zu, Peitao Liu

**Affiliations:** Institute of Physics & Optoelectronics Technology, Baoji University of Arts and Sciences, Baoji 721016, China; lixd@bjwlxy.edu.cn (X.L.); zuyanqing74@bjwlxy.edu.cn (Y.Z.); liupt@bjwlxy.edu.cn (P.L.)

**Keywords:** metalloporphyrin hydrides, mechanism, steric hindrance, homolysis and heterolysis, DFT, effective volume

## Abstract

Substituents at the *meso-*site of metalloporphyrins profoundly influence the hydrogen evolution reaction (HER) mechanism. This study employs density functional theory (DFT) to computationally analyze Ni^II^-porphyrin and its hydrides derived from tetrakis(pentafluorophenyl)porphyrin molecules, presenting stereoisomers in *ortho-* or *para*-positions. The results reveal that the spatial resistance effect of *meso*-substituted groups at the *ortho-* and *para-*positions induces significant changes in Ni-N bond lengths, angles, and reaction dynamics. For *ortho-*position substituents forming complex **I**, a favorable 88.88 Å³ spherical space was created, facilitating proton coordination and the formation of H_2_ molecules; conversely, *para-*position substituents forming complex **II** impeded H_2_ formation until bimolecular complexes arose. Molecular dynamics (MD) analysis and comparison were conducted on the intermediation products of **I-H_2_** and **(II-H)_2_**, focusing on the configuration and energy changes. In the **I-H_2_** products, H_2_ molecules underwent separation after 150 fs and overcame the 2.2 eV energy barrier. Subsequently, significant alterations in the spatial structure were observed as complex **I** deformed. In the case of **(II-H)_2_**, it was influenced by the distinctive “sandwich” configuration; the spatial structure necessitated overcoming a 6.7 eV energy barrier for H_2_ detachment and a process observed after 2400 fs.

## 1. Introduction

Hydrogen has garnered attention as a potential solution for reducing greenhouse gas emissions and promoting sustainability due to its clean, versatile, and renewable properties [1,2,3,4,5]. Among the various methods for hydrogen production, metallic porphyrins have emerged as promising catalysts owing to their unique electrochemical and photophysical properties [6,7,8,9,10,11,12] and their exceptional reactivity in energy-dependent small molecule activation [13]. Notably, the facile modification of porphyrins through substitution reactions at the *meso-*site [14,15,16,17,18], β-site [19,20,21], and axial coordination site [22,23] provides a means to control proton transfer ability, substrate accessibility to the active center, and product selectivity. Currently, several studies are investigating the hydrogen evolution reaction (HER) mechanism using metallic porphyrins [24,25,26]. The catalytic activity of these metal complexes relies on their ability to participate in proton-coupled electron transfer (PCET) processes, producing intermediates that donate hydrides to free protons and release hydrogen [27]. Furthermore, steric hindrance caused by *meso-*substituents in metal porphyrin hydrides has been found to affect the HER mechanism [28,29], and computational studies are underway to better understand this hemolysis and heterolysis process.

**Figure 1:**
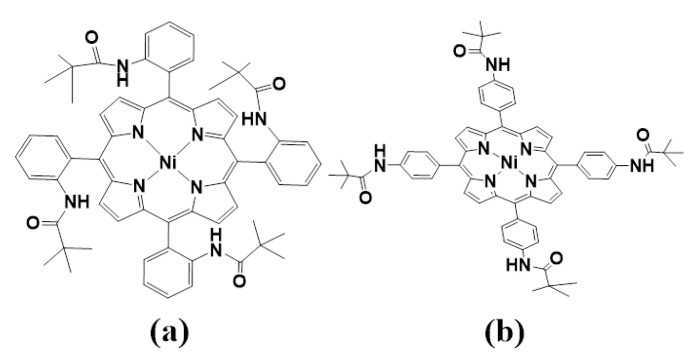
Structures of the **I** (**a**) and **II** (**b**) Ni^II^ porphyrin complexes.

**Figure 2:**
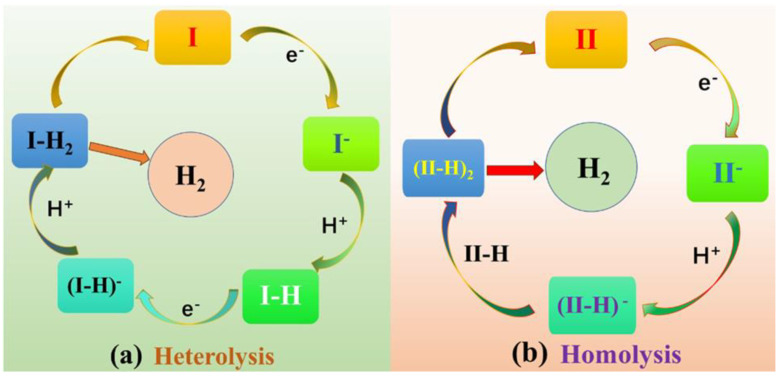
Catalytic HER mechanisms with I (**a**) and II (**b**).

Metalloporphyrin hydrides are pivotal in catalytic hydrogen production [30,31,32]. Previous studies have shown that the HER mechanism of metal porphyrin hydrides is significantly influenced by *meso-*substituted groups [33,34]. Recently, Cao et al. [33,35] conducted experimental investigations to determine how the size of *meso-*substituted groups directly affects the HER mechanism. Two *meso-*substituted groups were studied: phenyl groups connected by *para*-positions with amido moieties (Figure 1a) and substituents with the same *ortho*-positions (Figure 1b). The HER reaction process was characterized for each complex type (Figure 2). The results showed that, in the case of complex **I**, the bulky pivalamide group led to steric hindrance, forming two intermediate species: Ni^III^-H, which received one electron, and Ni^II^-H. Finally, heterolysis occurred to produce H_2_. Conversely, Ni^III^-H formed a bimetallic complex intermediate for complex **II** because of the absence of steric hindrance from the substituted group on the active site. The homolysis reaction then took place to produce H_2_. While it is known that the size of *meso-*substituted groups has a direct impact on the activity and reaction mechanism of the active center, there is a lack of formation of intermediates, and the protonation reaction, separation of H_2_ molecules from the system after formation, interaction with *meso-*position substituents, and charge distribution have not been studied in detail. Therefore, this study comprehensively investigates the effects of substituent steric hindrance on the reactions of complexes **I** and **II** and their intermediates. This investigation incorporates multiple aspects, including geometry, electron density distribution, atomic charge, the density of states, and molecular dynamics analysis. The findings of this study provide theoretical underpinnings for the future design and research of novel porphyrin catalysts.

## 2. Results and Discussion

### 2.1. Optimized Structure Analysis

The geometrical optimization of the **I** and **II** complexes and their related intermediate products was performed using the PBE0/def2-SVP method, as implemented in the Gaussian 09 software package. During the optimization process, the products were carried out at the lowest energy level of the high spin state, and all compounds were optimized to the local minimum of the potential energy surface. The structures of selected **I** and **II** complexes and their **I-H_2_** and **(II-H)_2_** compounds were visualized based on the optimized conformations, as shown in Figure S1. The analysis revealed that, in the complex **I**, where two pivalamido groups replaced the *meso*-site of the porphyrin ring, the planar structure of the porphyrin ring changed to a “saddle-like” structure due to spatial blockage, whereas in the **II** complex, the planar structure of the porphyrin ring was less perturbed. The results also show that the bond lengths of Ni^II^ ions and pyrrole N (N1) and pyridine N (N2) in **I** and **II** complexes underwent slight changes (Table 1). However, the corresponding bond lengths increased gradually when electrons and protons were combined, particularly when comparing the bond lengths of **I-H** and **II-H** complexes. This was primarily due to the spatial site resistance, which limited the further expansion of the Ni^II^-porphyrin ring and affected the bond length of Ni^II^-H in complex **I**. Similarly, this change also caused a corresponding significant change in the atomic charge, which will be discussed in Section 2.2

### 2.2. Atomic Charge Analysis

Atomic charges play a significant role in studying electrostatic interactions between molecules and predicting the properties of materials and their interactions with other substances. Accurate atomic charges can aid in designing ligands that interact favorably with proteins and other biological molecules and determine which atoms are likely to participate in catalytic reactions. However, due to the unobservable nature of the atomic charge and the lack of an objective and unique definition, numerous methods exist to calculate atomic charge [36,37]. The atomic dipole corrected Hirshfeld atomic charge (ADCH) method offers a more objective approach to atomic charge analysis, which is essential for predicting chemical reactivity, designing new materials, and understanding electrostatic interactions in biological systems. The ADCH method defines the atomic charge (Equation (1)) as a weighted sum of electron density contributions from neighboring atoms:

(1) $$q_{A}=\;-\int w_{A}(r)∆\rho (r)dr$$

where $∆\rho \left(r\right)=\rho \left(r\right)-\sum_{A}\rho_{A}^{0}$(r), $w_{A}(r)$=$\frac{\rho_{A}^{0}(r)}{\sum_{A}\rho_{A}^{0}(r)}$, where$\;\sum_{A}\rho_{A}^{0}$(r) represents the electron density of all atoms in the free state; ∆ρ(r) represents the deformation density, which shows the variation in the electron density during the chirality process after the atoms formed molecules; and $w_{A}(r)\;$is the A-atom weight function, defined as the region in the whole real space belonging to the A-atoms. However, the Hirshfeld charge data are generally small [38], and the dipole moment and electrostatic potential are poorly reproducible [39], mainly because the influence of the atomic dipole moment in the calculation process is neglected. To address this issue, Lu Tian [40] proposed the ADCH method, which defines the atomic dipole moment ($\mu_{A}$) as:

(2) $$\mu_{A}=\;-\int w_{A}(r)∆\rho (r)(r-r_{A})dr$$

In this method, the Hirshfeld charge of each atom and its$\;\mu_{A}$ are calculated first, and then each $\mu_{A}\;$is expanded into the calibrated positive charge of the surrounding atoms according to Equation (3).

(3) $$\mu_{A}=\sum_{B}∆q_{AB}r_{B}$$

$∆q_{AB}$ denotes the calibrated positive charge of the$\;\mu_{A}\;$of the unfolded A atom on the B atom. Finally, after unfolding the$\;\mu_{A}\;$of all atoms into the correctional charge and then accumulating it to the original Hirshfeld charge, the ADCH charge is obtained.

To further analyze the changes in atomic charges or fragment charges of Ni, N1, and N2 atoms, as well as their *meso-*site substituents in complexes **I** and **II** and their intermediate states throughout the entire reaction process, the ADCH method was employed for analysis at each step. The corresponding analysis results are presented in Table 2. The table shows that the *up*-Sub fragment in complex **I** carried a charge of −0.009 a.u., while the *down*-Sub fragment bore a charge of 0.008 a.u. This indicates an uneven charge distribution due to steric hindrance imposed by the substituent at the *meso-* position. This variation was also reflected in the charge distribution of nitrogen atoms in the porphyrin ring. Pyrrole N1 had a charge of 0.143 a.u., whereas, on pyridine N2, the charge was −0.179 a.u. In complex **II**, the fragment charges carried by the substituents at the *meso-*position were equal, and the charges on pyrrole N1 and pyridine N2 were also identical. Moving on to the analysis of the intermediate complexes **I^−^** and I**I^−^**, in Table 2, it can be observed that after receiving an electron, the charges carried by the substituents at the *meso-*position in complexes **I^−^** and **II^−^** were both negative. Notably, the negative charge in complex **I^−^** was more significant than in II^−^ by approximately 0.015 a.u. Conversely, the charges on pyrrole N1 and pyridine N2 were opposite. Simultaneously, the charges on the Ni ion in complexes **I^−^** and **II^−^** were identical. From these changes, it can be inferred that in complex I**^−^**, due to the greater negative charge carried by the substituent at the *meso-*position compared to the fragment in **II^−^**, a negative electric field was expected to form around complex **I^−^**, attracting protons into the coordination center and enhancing the proton-accepting capability.

Upon further proton coordination with complexes **I** and **II**, it was observed that the charges carried by the substituents at the *meso-*position became positive, while the charges on pyrrole N1 and pyridine N2 essentially remained unchanged and hostile. The primary alteration was detected in the central coordinating metal Ni^II^ ion. In the subsequent process, due to the steric hindrance exerted by the substituent at the *meso-* position in complex **I-H**, further electron acceptance led to the formation of the intermediate complex **(I-H)^−^**. In this intermediate state, the fragment charges carried by the substituent at the *meso-* position and pyrrole N1 and pyridine N2 all became negative. This change indicated the electron-donating capability of the *meso*-sited substituent, simultaneously creating a spatial environment with a strong proton-accepting ability around complex (**I-H**). This environment provided a conducive space for the further binding of protons to form hydrogen molecules. Drawing on earlier research findings and comparing the process of stable H_2_ formation in complex **II-H** [33], it was inferred that, since the *meso*-sited substituents in complex **II-H** carried positive charges, this might pose challenges for the formation of a stable **II-H_2_** complex. This analysis suggests that **II-H** is less likely to form a stable diatomic complex under these conditions, which would hinder the formation of H_2_ molecules.

Alternatively, an ADCH analysis was performed on the diatomic complex **(II-H)_2_**, derived from the dimerization of complex **II-H**. The examination unveiled notable discrepancies in the fragment charges borne by pyrrole N1, pyridine N2, and the *meso*-sited substituent. Furthermore, variations in the charges of the central ligand Ni^II^ were observed. The comprehension of these observed changes is presently in a preliminary exploration stage, and a more comprehensive investigation will be undertaken in subsequent studies.

### 2.3. Fragment Orbital Interaction Analysis

**Figure 3:**
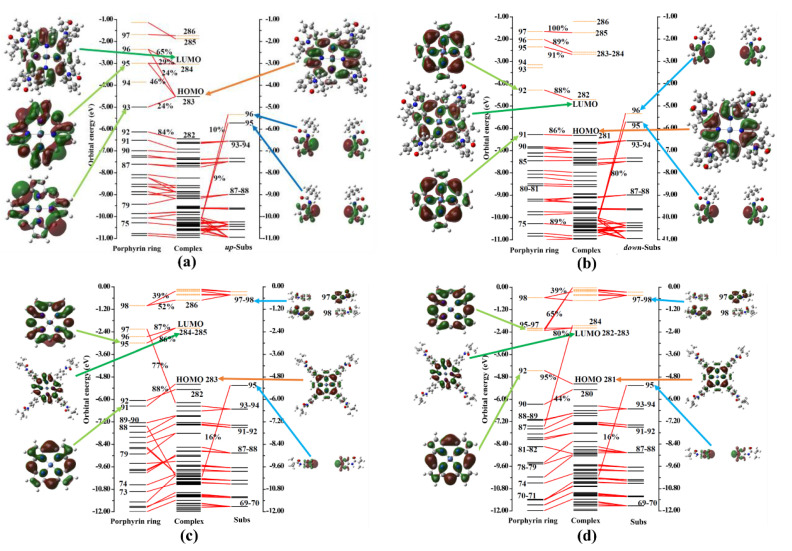
Fragment orbital interaction diagram of porphyrin rings, *up-*substituents (*up*-subs), and *down-*substituents (*down-*subs). Black solid and red dashed bars correspond to occupied and unoccupied MOs. (**a**,**c**) indicate the major contribution of alpha MOs of the porphyrin ring and *up-*sub fragments to the **I**. (**b**,**d**) indicate the major contribution of beta MOs of the porphyrin ring and *down-*sub fragments to the **II**. The orbital compositions were evaluated using the Mulliken method. Note: *up*-substituent indicates a *para*-position substituent close to a proton; *down*-substituent indicates a distant substituent group.

Based on the previous analysis of the atomic charges and fragment charges of complexes **I** and **II**, as well as their corresponding reaction intermediates, it is evident that the bulky pivalamide group positioned at *ortho* and *para* sites had a significant impact on the charge distribution of the porphyrin ring and its Ni ions. This impact is primarily attributed to the interaction of molecular orbitals derived from the substituted fragment charges. To further delve into the interaction between substituted fragment molecular orbitals and the molecular orbitals of the porphyrin ring, this study employed the General Charge Decomposition Analysis (GCDA) method proposed by Dapprich and Frenking [41], as refined by Lu Tian [42]. The results of the analysis are presented in Figure 3 and Figure S1.

As depicted in Figure 3, in both complexes **I** and **II**, the orbitals constituting their Highest Occupied Molecular Orbital (HOMO) and Lowest Unoccupied Molecular Orbital (LUMO), be they alpha or beta molecular orbitals, were primarily composed of the molecular orbital components of the HOMO and LUMO of the porphyrin ring. The molecular orbital components of the substituent groups at the *meso*-site did not prominently appear in the corresponding orbitals. Additionally, in conjunction with Figure S2, it is evident that when complexes **I** or **II** further interacted with electrons or protons to form intermediates, their respective HOMO and LUMO molecular orbitals remained composed of the frontier molecular orbitals of the Ni metal porphyrin ring. The frontier molecular orbital components of the corresponding up-subs or down-subs primarily constituted molecular orbitals lower than HOMO or higher than LUMO.

It was observed that the substituent groups at the *meso-*position did not significantly participate in the formation of chemical bonds throughout the entire reaction process, but rather acted as steric hindrances.

### 2.4. Steric Hindrance Analysis

**Figure 4:**
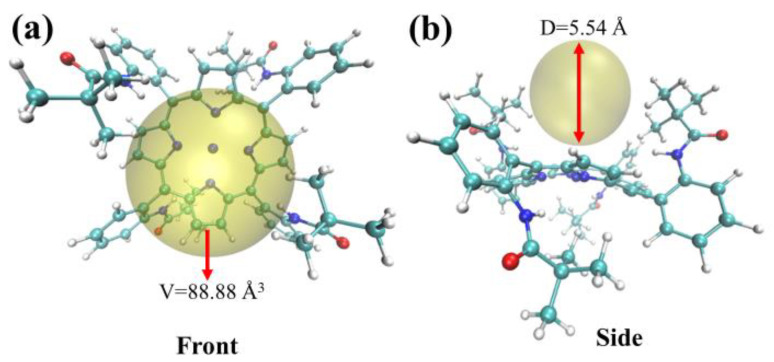
Steric hindrance analysis of complex **I**. Note: Yellow sphere shows an unoccupied region inside of complex **I.** (**a**) Front (**b**) Side.

This paper aimed to investigate the impact of steric hindrance from bulky pyramidal group substituents on the active center (Ni^II^) and to understand the mechanism of steric effects on the heterolysis reaction in the formed **I-H** intermediate. To this end, we employed the Multiwfn software to analyze the geometric structure of the **I-H** intermediate under a triplet spin state. The resulting structure was analyzed using the visualization software VMD, and the analysis results are presented in Figure 4.

As shown in Figure 4, the yellow sphere represents the unoccupied space in the **I-H** complex, surrounded by two pivalamido groups. This region had an average diameter of 5.54 Å and a corresponding volume of 88.88 Å^3^. Protons with diameters of approximately 10^−5^Å were able to freely enter and exit this region, indicating that they could react with the intermediate **(I-H)^−^**. However, **(I-H)^−^** was unable to form a bimetallic complex or undergo a homolysis reaction. These findings demonstrate that the size of the substituent directly affects the reaction mechanism.

In conclusion, this study sheds light on the influence of steric hindrance on the heterolysis reaction in the formed **I-H** intermediate. It provides a reasonable explanation for the observed reaction mechanism.

### 2.5. Density-of-State Analysis

**Figure 5:**
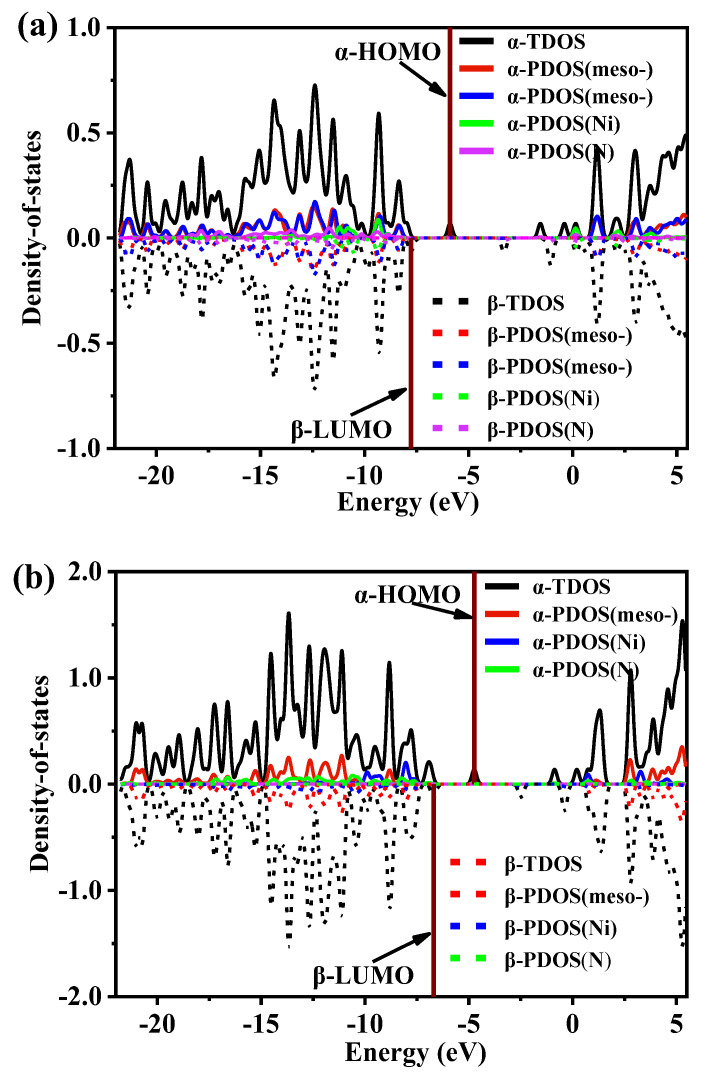
Density-of-state (DOS) map and MOs degeneracy of the (**a**) **I** and (**b**) **II** complexes. The location of the HOMO is a wine-colored vertical line.

The density of states (DOS) maps visualize the energy distribution of molecular orbitals (MOs) in a chemical system [43], and the value of the DOS curve reflects the number of MOs at the corresponding energy per unit energy interval. In Figure 5, the total DOS (TDOS) maps for all MOs of **I** and **II** and the partial DOS (PDOS) maps contributed by various groups of MOs are plotted. In plotting the DOS and PDOS, the α and β MOs were plotted separately, as both systems of the two complexes used the open-shell layer type. All the α Mos curves are located in the upper half of the graph box (marked by solid lines), and the β MOs curves are in the lower half of the graph box (marked by dotted lines). When the α and β MOs are in perfect symmetry, it means that the electrons in the α and β orbitals are not spin-hybridized in the same energy region, and vice versa, spin polarization occurs.

As can be seen from the curve changes in Figure 5, the TDOS curves for the α and β MOs of each complex were stored in an asymmetric state, mainly due to the different distributions of electrons in the α and β orbitals. This result is significant for the highest energy molecular orbital (highest occupied orbital HOMO). As seen in Figure 5, there was a significant difference in the HOMO energies of the α and β MOs in the **I** and **II** complexes, and the HOMO energy in **I** was lower. To further analyze the effects of various groups in the various complexes on TDOS, the meso-site substituent (abbreviated as *meso-*), the pyridine N(N) on the porphyrin ring, and the central metal Ni^II^ ion were analyzed separately and identified by PDOS. As seen in Figure 5, the *meso-*site substituents in the individual complexes contributed significantly to the overall orbital energy level. However, differences remained in the **I** complex due to the presence of two different spatially distributed substituent types, as seen from the PDOS curves in Figure 5a (in red and blue lines). The additional Ni^II^ and the four N atoms contributed the most to the molecular front orbitals of HOMO and LUMO. This indicated the location of the catalytically active center.

### 2.6. Molecules Dynamics Analysis

**Figure 6:**
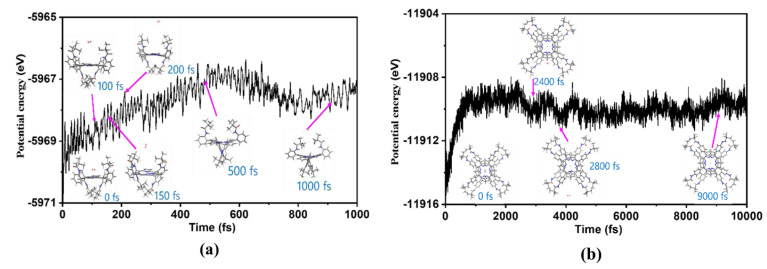
MD simulations depict the dissociation process of H_2_ molecules from compounds **I-H_2_** (**a**) and **(II-H)_2_** (**b**) at 298.15 K, employing the PBE/GFN1-xTB level of theory. The graph represents the relationship between reaction time and the system’s total energy (electronic energy).

To investigate the dissociation dynamics of H_2_ molecules from complexes **I-H_2_** and **(II-H)_2_** and to compare the reaction barrier magnitudes, this study utilized the molecular dynamics (MD) module within CP2K to simulate the dissociation process. The simulation maintained a reaction temperature of 298.15 K, and the MD analysis results are presented in Figure 6. For the **I-H_2_** system, H_2_ molecules exhibited dissociation from the coordination entity at approximately 150 fs, extending the simulation time to around 500 fs. A notable alteration in the spatial distribution of *meso*-sited substituents was observed compared to the initial structure. Mainly, amino acid substituents exhibited increased proximity, leading to a gradual contraction of the central region formed by amino acid substituents, albeit without a significant alteration in the overall energy of the complex system.

In contrast, under identical simulation conditions for the **(II-H)_2_** system, at 150 fs, H_2_ molecules remained within the coordination field formed by two Ni**^II^** ions. Only after extending the simulation time to 2400 fs did H_2_ molecules dissociate from the complex system. The “sandwich” structure in this system, comprising two complexes **II**, was found to tightly constrain the spatial configuration, impeding the dissociation of H_2_ molecules. In Figure 6b, it is evident that, after 240 fs, the total energy of the entire system underwent a relatively modest change. This change implies that, within the initial 2400 femtoseconds, the overall complex system was unstable. However, due to the “sandwich” structure, H_2_ dissociation was restricted, hindering the anticipated reduction in system energy.

Moreover, upon comparing the energy variations depicted in Figure 6a,b, we observed that the total energy for the **I-H_2_** complex was −5570.6 eV initially and −5568.4 eV at 150 fs; this indicates that the H_2_ molecule needed to surpass a reaction energy barrier of 2.2 eV to detach from the complex system. Conversely, as shown in Figure 6b, the initial total energy for the **(II-H)_2_** complex was −11,916.5 eV, and after 2400 fs, it increased to −11,909.8 eV. In this scenario, the H_2_ molecule encountered a higher reaction energy barrier of 6.7 eV for detachment. Notably, the **I-H_2_** system exhibited a more favorable condition for separating H_2_ molecules, which requires overcoming a lower reaction energy barrier.

## 3. Computational Methods

This paper details the computational methods employed for optimizing the geometries and conducting single-point calculations for complexes **I** and **II**. All calculations were performed using the Gaussian 09 package [44]. Geometry optimization utilized the hybrid functional PBE0 [45] in conjunction with the def2-SVP basis set [46], while single-point calculations employed the def2-TZVP basis set. Chloroform was selected as the solvent for the simulation. To enhance the congruence between calculated results and original data, the simulated NMR spectra were compared with experimental spectra outlined in reference [33] (Figure S3). This comparison aimed to validate the appropriateness of the selected theoretical methods and basis sets during the calculation process, indicating the effective simulation of the transformation process using the current theoretical approach. The optimized structures of **I** and **II** obtained at the def2-TZVP level are presented in Figure S2, accompanied by the calculated bond lengths listed in Table 1 and corresponding experimental values. The results from the PBE0/def2-SVP calculations align with the experimental values from reference [33].

To further analyze the geometric structure of steric hindrance in the electron-receiving and protonation processes, as well as the relative electron distribution density changes, the intermediate products were optimized at the def2-SVP level. Their geometric optimization and analysis were carried out using PBE0/def2-SVP. Single-point calculations were conducted at the TZVP level, and the optimized structures of the intermediate products were identified as local minima without imaginary frequencies.

The electronic structure analyses were conducted using the Multiwfn 3.8 (dev) software [47], with isosurface maps of multiple orbitals and real space functions generated using the Visual Molecular Dynamics (VMD) program [48]. The files exported from Multiwfn were employed as an input for VMD to generate the plots.

To further analyze and compare the changes of the *meso-*substituted groups and binding energies of **I** and **II** complexes during the process of forming the intermediate products **I-H_2_** and **(II-H)_2_**, where H_2_ dissociates from the active site of the coordinating metal ion to become an independent H_2_ compound, density functional theory (DFT) calculations were conducted. The Perdew–Burke–Ernzerh exchange–correlation functional for complexes (PBE) [49,50], along with dispersion correction (DFT-D3), was employed using the Quickstep module of the CP2K program [51,52]. Molecular dynamics studies were conducted for the **I-H_2_** and **(II-H)_2_** complexes. We constructed a three-dimensional periodic framework structure to encapsulate the **I-H_2_** and **(II-H)_2_** complexes. For the framework structure corresponding to the **I-H_2_** complex, a = b = 17.8 Å and c= 15.5 Å, and for the framework structure corresponding to the **(II-H)_2_** complex, a = b = 26.0 Å and c = 15.3 Å. Simulations were performed at 298.15 K using PBE/GFN1-xTB for 1000 fs of **I** and 10,000 fs of **II** complexes. The canonical sampling through velocity rescaling (CSVR) ensemble in the NVT (number, volume, temperature) ensemble was employed.

## 4. Conclusions

In this paper, a density functional theory (DFT) approach was employed on the hybrid functional PBE0 in conjunction with the def2-SVP basis set for the analysis of Ni^II^-porphyrin containing different *meso*-site substituents. The analysis of Ni^II^-porphyrin and its corresponding hydrides containing different *meso-*site substituents was carried out on the hybrid functional PBE0 in conjunction with the def2-SVP basis set to understand the role of steric hindrance in the HER reaction mechanism. The structural optimization of the **I** and **II** complexes revealed that the four Ni-N bond lengths were equal, but I was slightly shorter than **II** by 0.01 Å. Upon further binding to the proton, the corresponding Ni-N bond lengths of the two complexes varied more significantly, while the bond lengths of the Ni^II^-H bonds also differed significantly. In addition, the analysis of ADCH showed that the charges of the substituents at opposite positions were also different, which was the main reason for the change in bond length. In addition, the volume of the space surrounded by the two substituents and the porphyrin ring in the I complex was investigated, and it was found that the effective diameter and importance of the area allowed the proton to move freely, but prevented the formation of the bimetallic complex, which led to the heterolysis reaction.

Further analysis by DOS revealed that, in both the **I** and **II** complexes, the *meso*-site substituent contributed to the entire molecular orbital, especially the non-HOMO orbital. Still, Ni^II^ and the N atom contributed the most to the HOMO. After GCDA analysis of the **I** and **II** complexes and their corresponding intermediates, we found that the HOMO of the porphyrin ring and the alpha or beta molecular orbitals of LUMO were still composed of the frontier molecular orbitals of Ni^II^-porphyrin. The frontier molecular orbital components of the corresponding up-subs or *down-*subs did not participate in the protonation process and mainly played a steric hindrance role. Furthermore, MD analysis of the **I-H_2_** and **(II-H)**_2_ systems formed after protonation was conducted at 298.15 K. The results showed that, for the **I-H_2_** system, H_2_ molecules separated from complex **I** at about 150 fs, and the reaction energy barrier of 2.2 eV was overcome. As for the corresponding **(II-H)**_2_ system, because of its “sandwich” system, its tight spatial structure limited the separation of H_2_ molecules to 2400 fs. H_2_ molecules could be separated from the complex design and overcome the reaction energy barrier of 6.7 electron volts. Complex **I** was more conducive to the formation of protonation reactions and the separation of H_2_ molecules. Overall, after accessing the *meso*-site of the porphyrin ring with different stereoisomerisms and a bulkier substituent, the HER reaction was still able to proceed in the active Ni^II^ ion center. However, the reaction mechanism underwent a hemolysis and heterolysis type of distinction.

## Figures and Tables

**Table 1:** The selected bond lengths of **I, II**, and intermediate complexes calculated at the PBE0/def2-SVP level of theory.

Complexes	Distance (Å)		
	Ni-N1 ^a^	Ni-N2 ^b^	Ni-H
**I** **I^−^** **I-H** **(I-H)^−^** **II** **II** ^−^ **II-H**	1.94 1.94 2.02 1.97 1.95 1.95 2.08	1.95 1.95 2.04 2.06 1.95 1.96 2.09	- - 1.72 1.41 - - 1.67

a: pyrrole N; b: pyridine N.

**Table 2:** The selected charges of **I, II**, and intermediate complexes calculated at the PBE0/def2-SVP level of theory.

Complexes	ADCH Charge (a.u.)				
	*up*-Sub ^a^	*down*-Sub ^b^	Ni	N1 ^c^	N2 ^d^
**I** **I^−^** **I-H** **(I-H)^−^** **I-H_2_** **II** **II** **II-H**	−0.009 −0.075 0.043 −0.003 0.016 0.012 −0.060 0.053	0.008 −0.080 0.030 −0.025 0.031 0.012 −0.059 0.058	0.135 0.087 0.233 0.138 0.251 0.397 0.087 0.266	0.143 −0.156 −0.135 −0.170 −0.177 −0.204 −0.165 −0.112	−0.179 −0.157 −0.200 −0.310 −0.307 −0.204 −0.165 −0.176

Note: ^a^ indicates that the substituent groups of *meso-ortho*-positions on the porphyrin ring of complex **I** were close to Ni-H bond (*up*-Sub). ^b^ indicates that the substituent groups of *meso-ortho*-positions on the porphyrin ring complex **I** were far away from Ni^II^-H bond (*down*-Sub). ^c^ pyrrole N. ^d^ pyridine N.

## Data Availability

The raw or processed data necessary to reproduce the findings presented in this study are available upon request. Interested parties may contact the corresponding author for access to the data.

## Supplementary Materials

The following supporting information can be downloaded: https://www.mdpi.com/article/10.3390/molecules29050986/s1. Figure S1: Optimized structure of **I, II, I-H_2_**, and **(II-H)_2_**; Figure S2: Fragment orbital interaction diagram of porphyrin rings, up-substituents (*up*-subs), and down-substituents (*down*-subs). Black solid and red dashed bars correspond to occupied and unoccupied MOs. Figure S3: Simulated Nuclear Magnetic Resonance Hydrogen (HNMR) Spectrum of (a) and (b) complexes **I** and refer to the intermediates of **I^−^, I-H** and **(I -H)^−^**; (c) and (d) complexes **I** and refer to the intermediates of **II^−^, II-H** and **(II-H)_2_**. Ref. [33] is cited in Supplementary Materials.
