# Supplementary material for: Unraveling Meso-Substituent Steric Effects on the Mechanism of Hydrogen Evolution Reaction in NiII Porphyrin Hydrides Using DFT Method
Source: Molecules. 2024 Feb 23;29(5):986. doi: 10.3390/molecules29050986 (PMC10934329; doi:10.3390/molecules29050986)
Supplement: Supplementary file 1 [file molecules-29-00986-s001.zip › molecules-2809724-supplementary.pdf]

Supplementary:

Unraveling Meso-Substituent Steric Effects on the Mechanism of Hydrogen Evolution Reaction in Ni^{II} Porphyrin Hydrides using DFT Method

Xiaodong Li[a], Ailing Feng[a], Yanqing Zu[a], Peitao Liu[a], Meimei An[b]

[a] Institute of Physics & Optoelectronics Technology, Baoji University of Arts and Sciences, Baoji, 721016, China

[b] College of History Culture and Tourism, Baoji University of Arts and Sciences, Baoji, 721016, China

Abstract: Substituents at the meso-position of metalloporphyrins profoundly influence the hydrogenation reaction (HER) mechanism. This study employs Density Functional Theory (DFT) to computationally analyze NiII-porphyrin and its hydrides derived from tetrakis(pentafluorophenyl)porphyrin molecules, featuring stereoisomers in *ortho-* or *para*-positions. Analytical tools were applied to unravel the reaction dynamics, including Atomic charge analysis, Fragment orbital interaction analysis, Steric hindrance analysis, Density-of-state analysis, and Molecular dynamics (MD). The spatial resistance effect of *meso*-groups at and positions induced significant changes in Ni-N bond lengths and angles, especially post-protonation, creating a favorable 88.88 Å³ spherical space in complexes **I**. This space facilitates proton coordination, stabilizing the H_2 molecule. Conversely, complexes **II** impede H_2 formation until bimolecular complexes arise. The *meso*-position substituent, mainly uninvolved in chemical bond formation, acts as a spatial barrier, as demonstrated by Density-of-state. MD analysis of **I-H**$_2$ and **(II-H)**$_2$ systems revealed H_2 detachment from the ligand body in the **I-** system at 150 fs. Subsequent protonation reactions or losses after 500 fs underscore the dynamic behavior. In **(II-H)**$_2$, the spatial structure, influenced by the "sandwich" configuration, necessitates overcoming a 6.7 eV energy barrier for H_2 detachment after 2400 fs.

Keywords: Metalloporphyrin hydrides; mechanism; steric hindrance; homolysis and heterolysis; DFT; effective volume

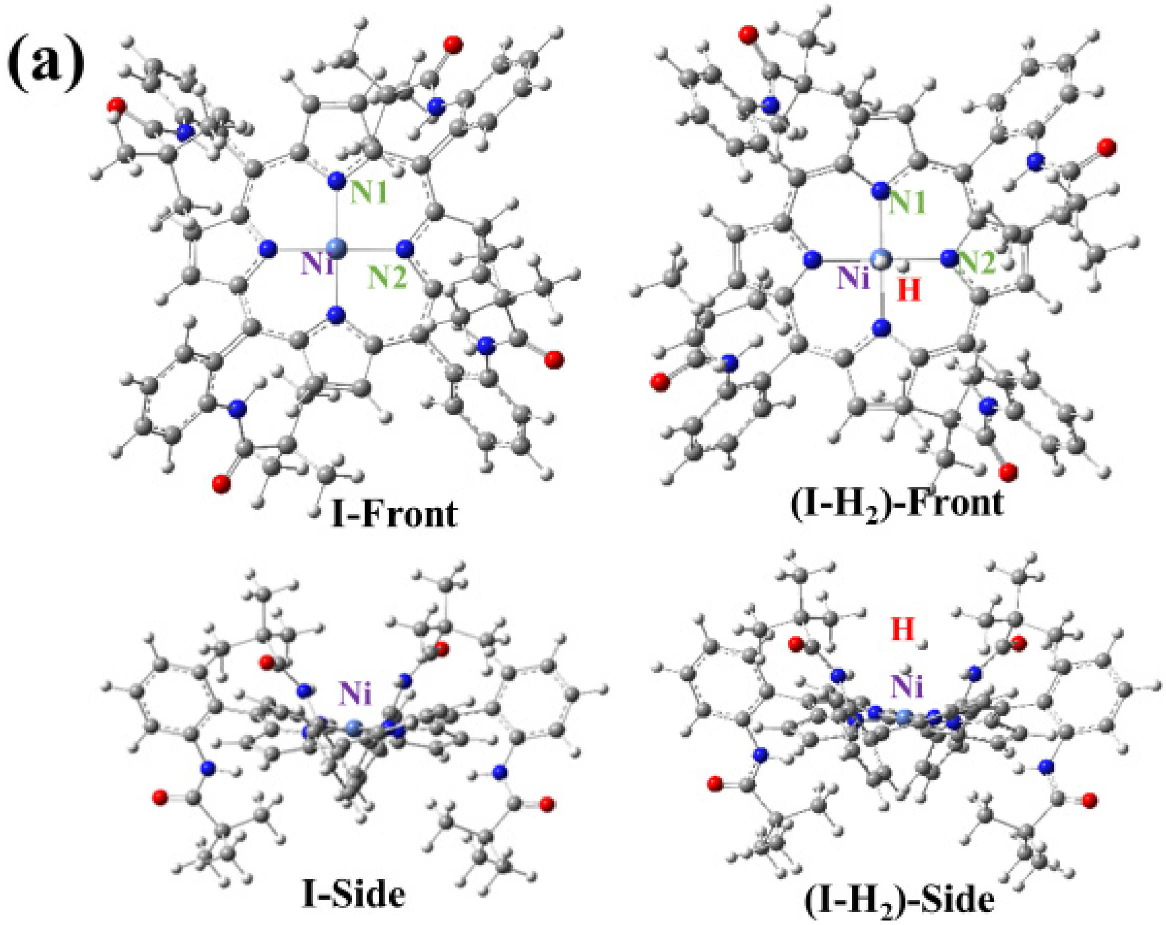
(a)
N1
Ni
N2
I-Front
N1
Ni
H
N2
(I-H2)-Front
Ni
I-Side
H
Ni
(I-H2)-Side

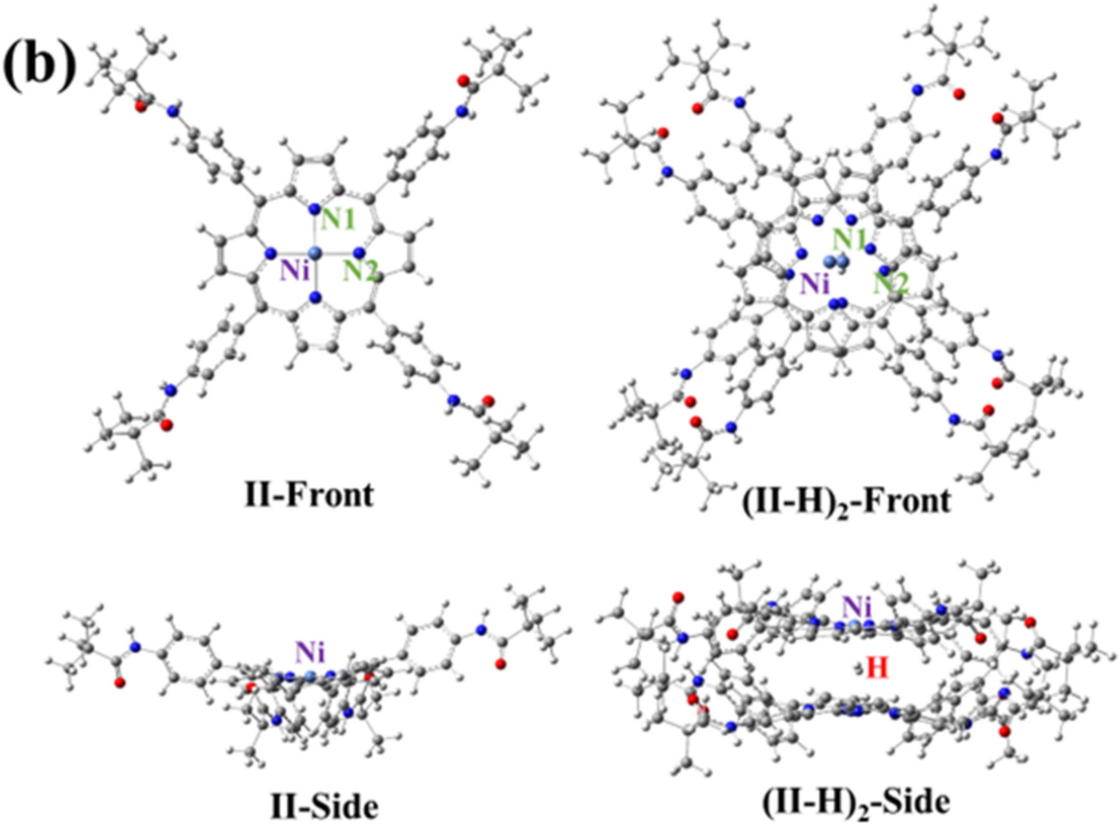

Figure S1. Optimized structure of Ⅰ, Ⅱ, Ⅰ-H_2 and (Ⅱ-H)$_2$

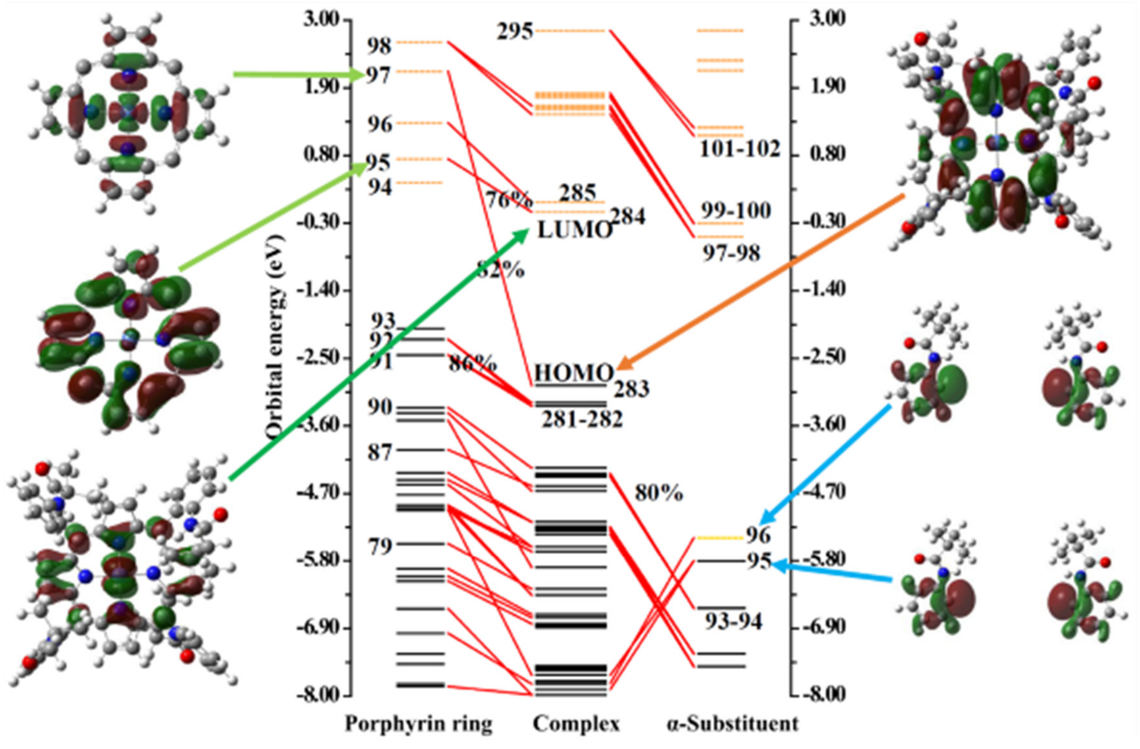

$(I^-)_\alpha$

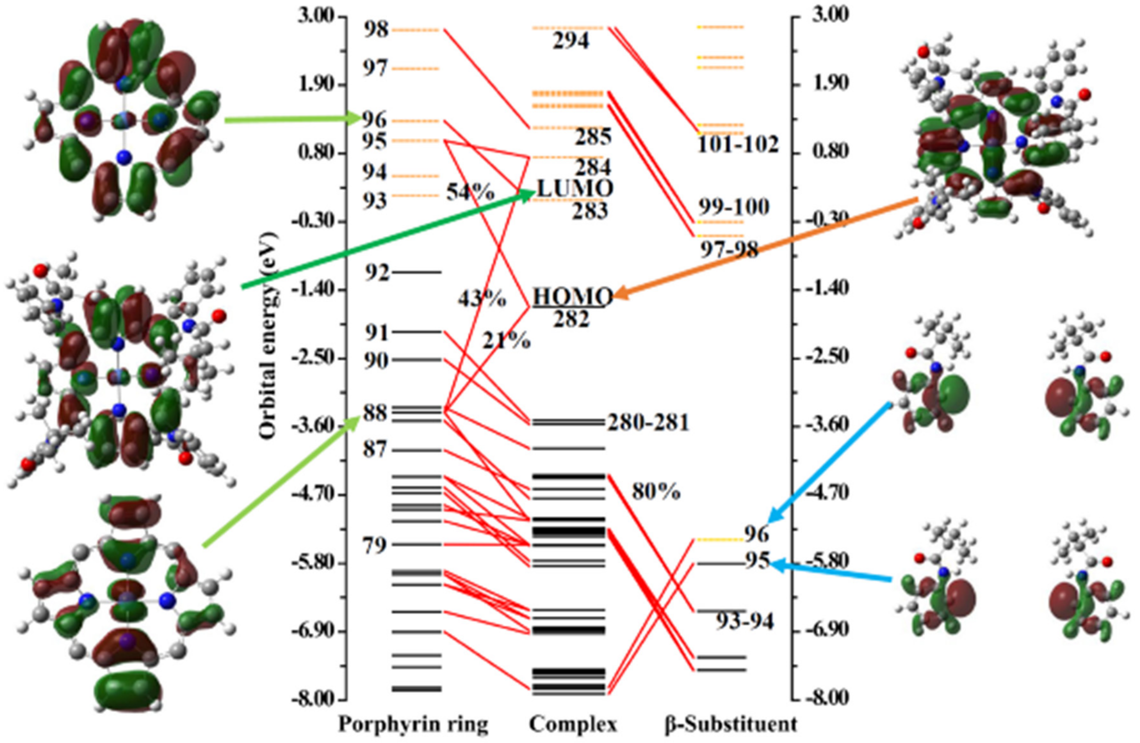

(I^{-})$_{\beta}$

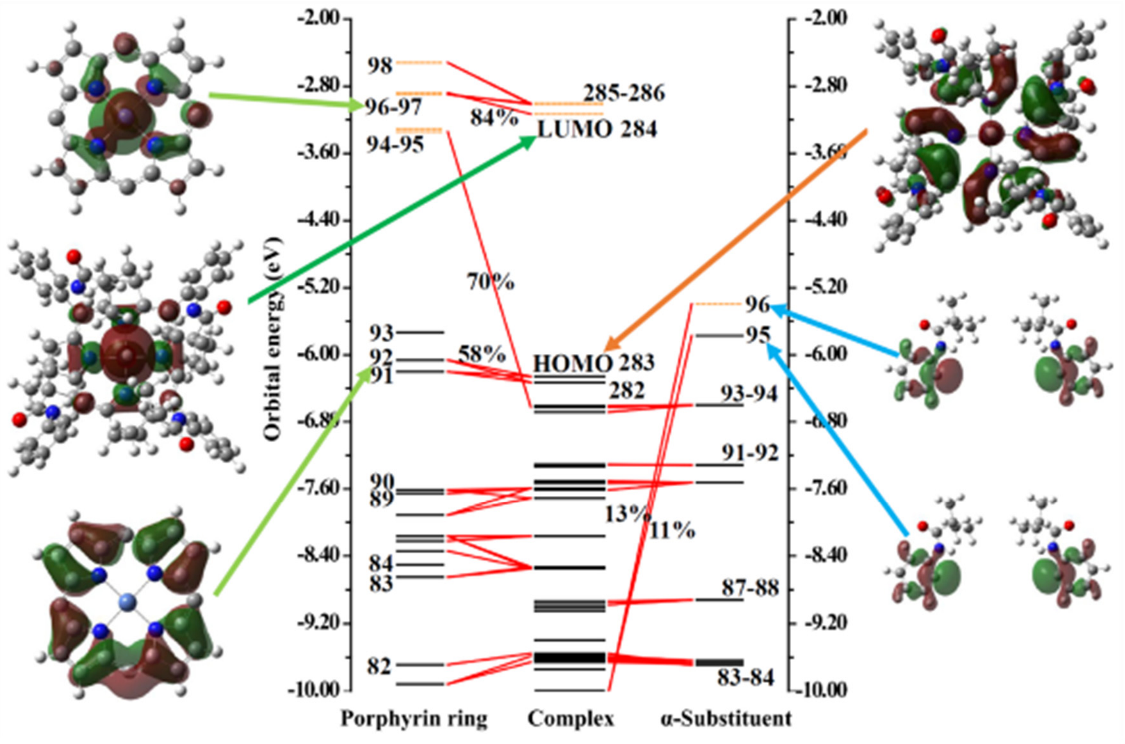

(I-H)$_{\alpha}$

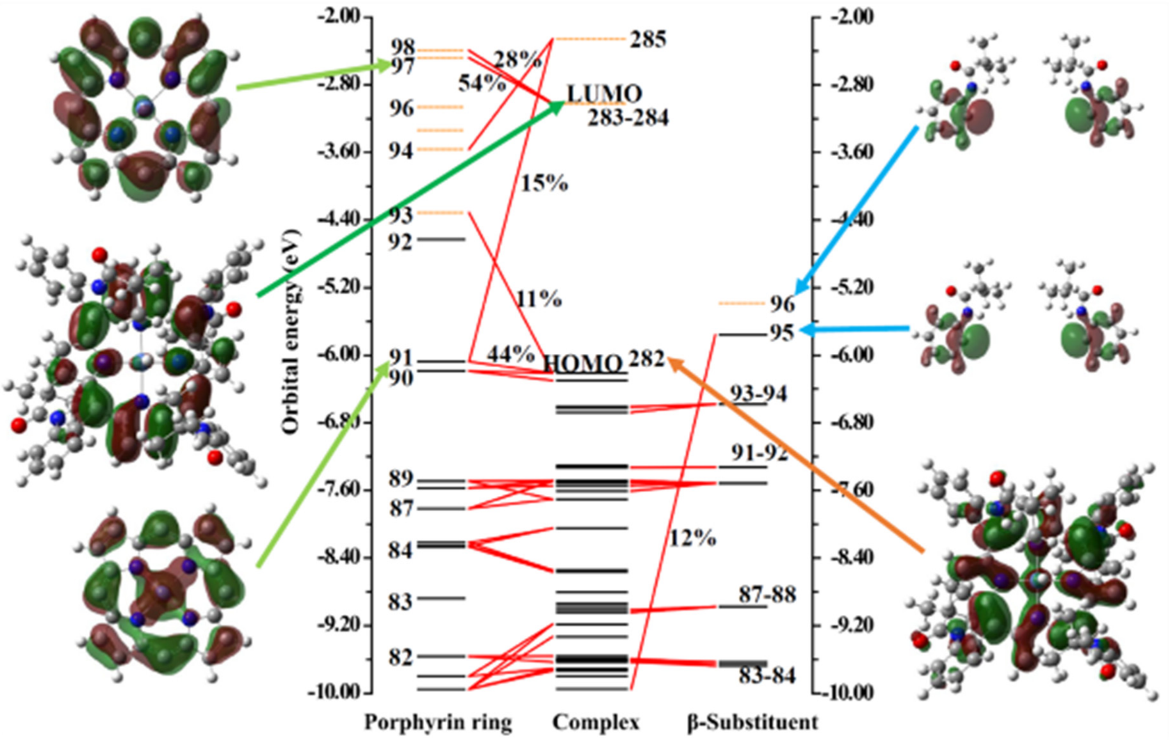

$(\text{I-H})_{\beta}$

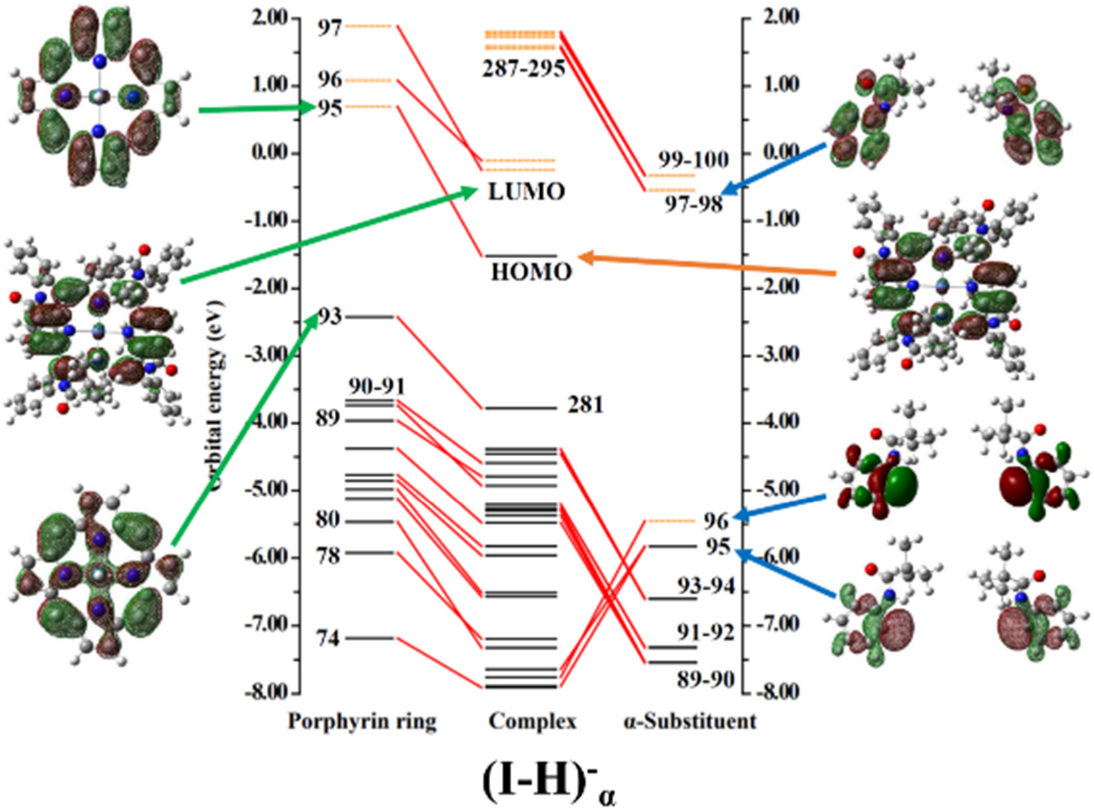

$(\text{I-H})^{-}_{\alpha}$

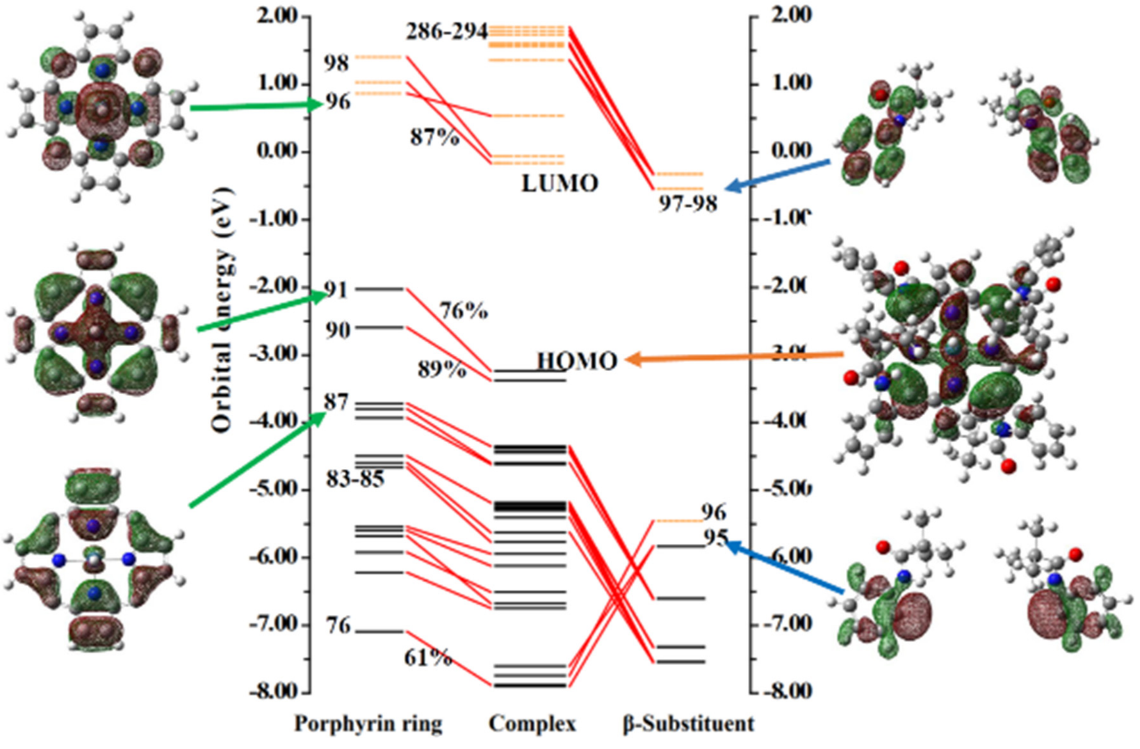

(I-H)$^-_{\beta}$

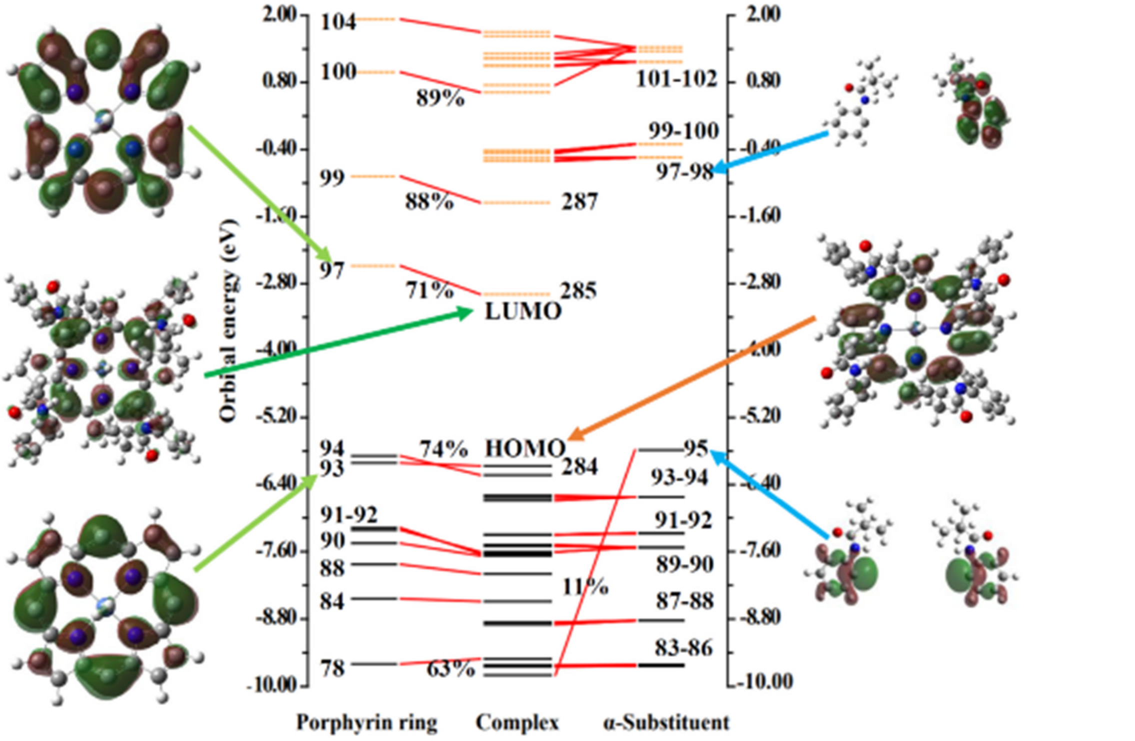

(I-H$_2$)$_{\alpha}$

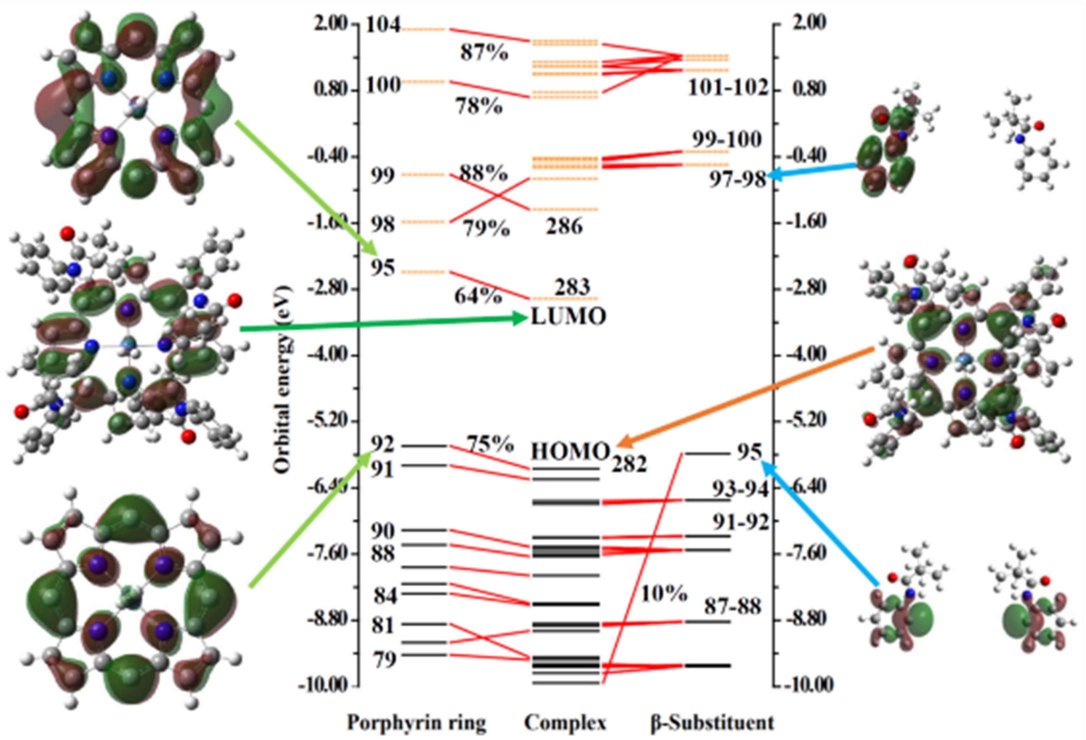

$(I\text{-}H_2)_\beta$

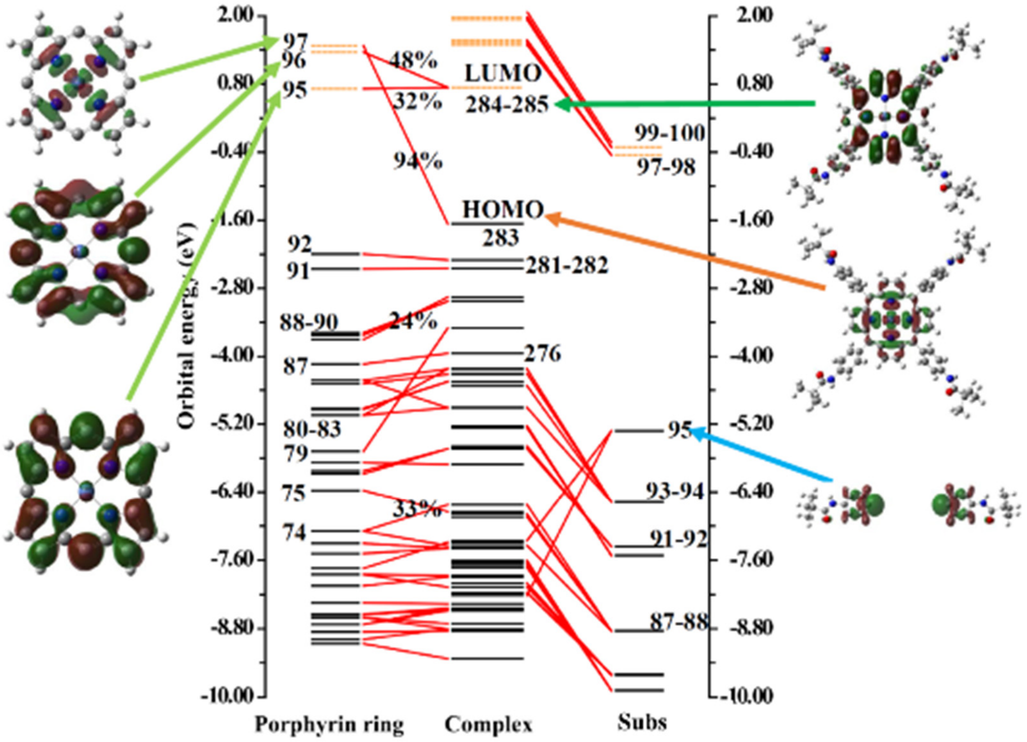

$(II^-)_\alpha$

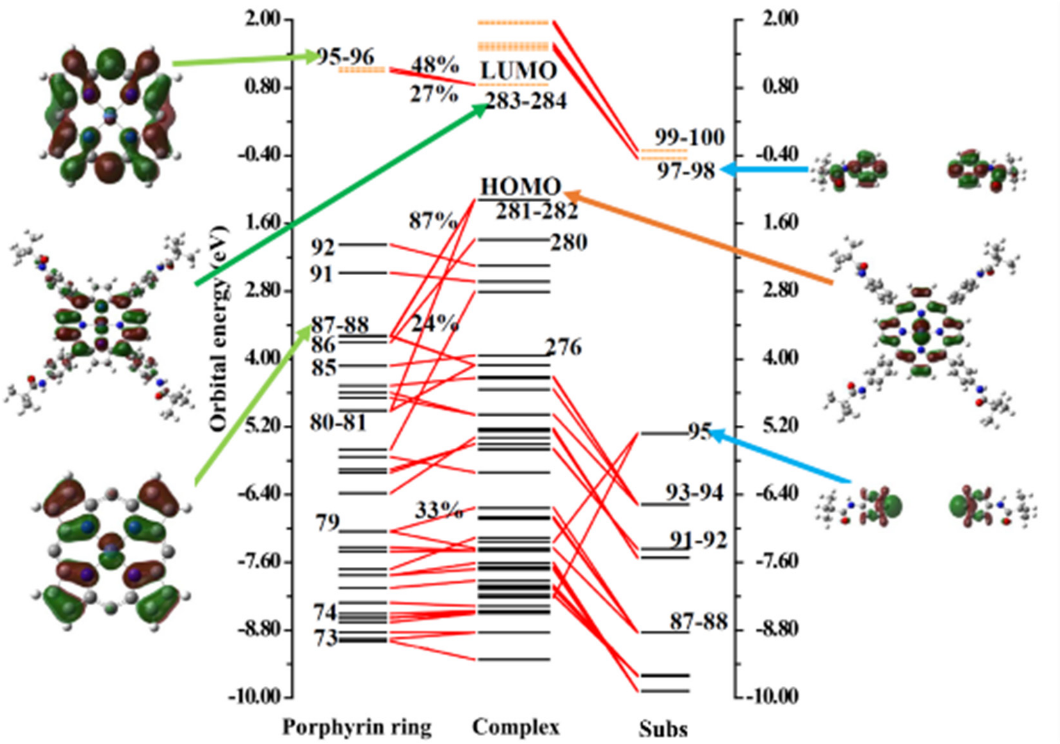

$(II^{-})_{\beta}$

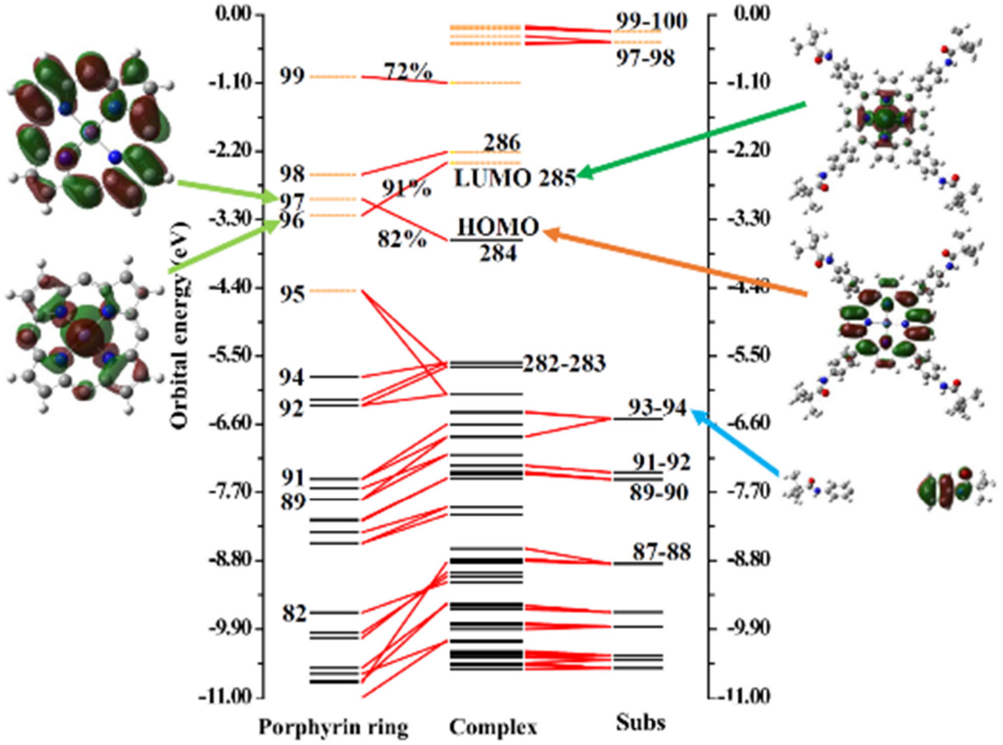

$(II\text{-}H)_{\alpha}$

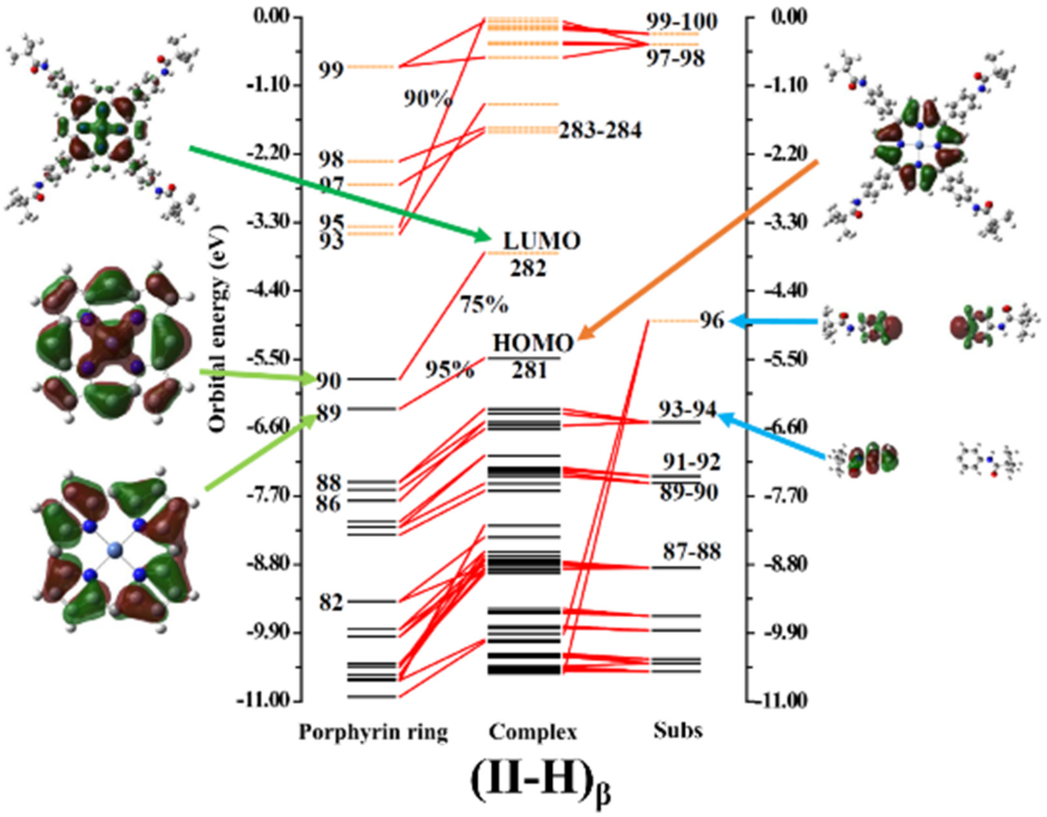

Figure S2. Fragment orbital interaction diagram of porphyrin rings, up-substituents (*up*-subs) and down-substituents (*down*-subs). Black solid and red dashed bars correspond to occupied and unoccupied MOs.

Note： *up*-substituent indicates a para-positions substituent close to a proton; *down*-substituent indicates a distant substituent group.

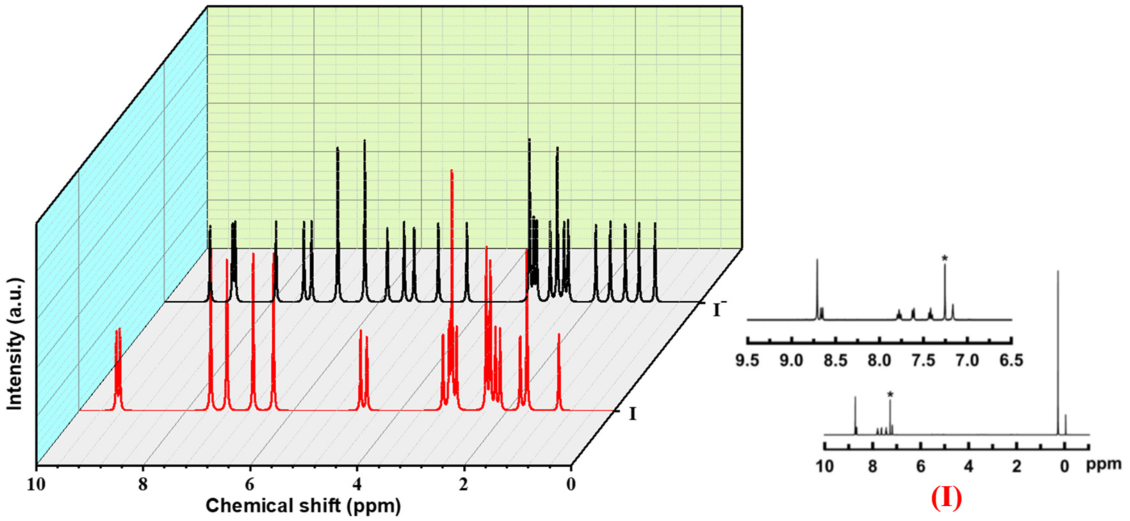

(a)

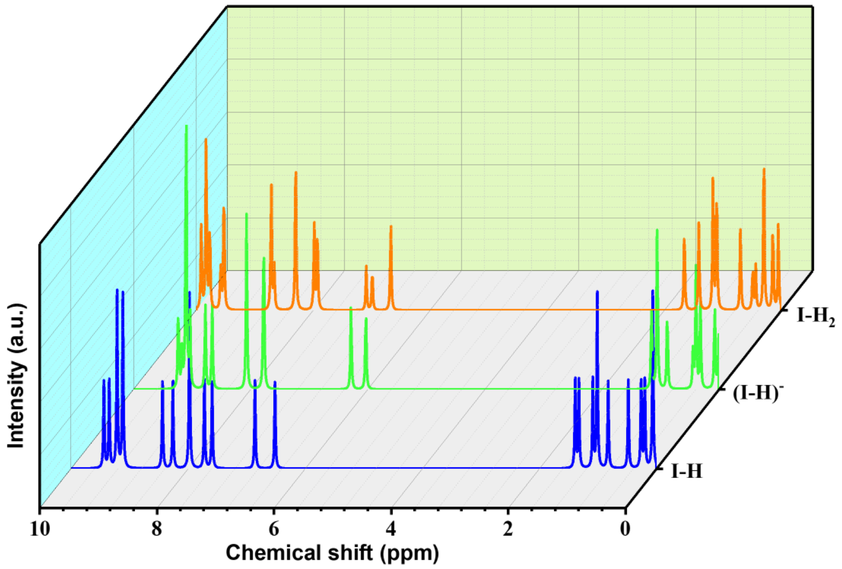

Intensity (a.u.)
I-H$_2$
(I-H)$^-$
I-H
10
8
6
4
2
0
Chemical shift (ppm)

(b)

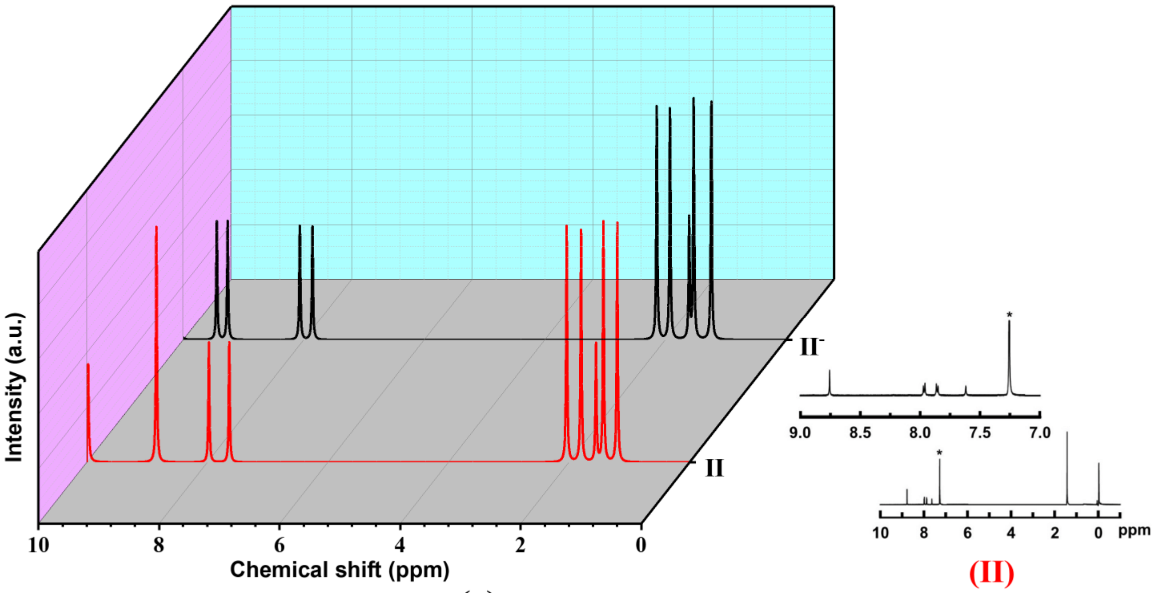

Intensity (a.u.)
II$^-$
II
10
8
6
4
2
0
Chemical shift (ppm)
9.0
8.5
8.0
7.5
7.0
10
8
6
4
2
0
ppm
(II)

(c)

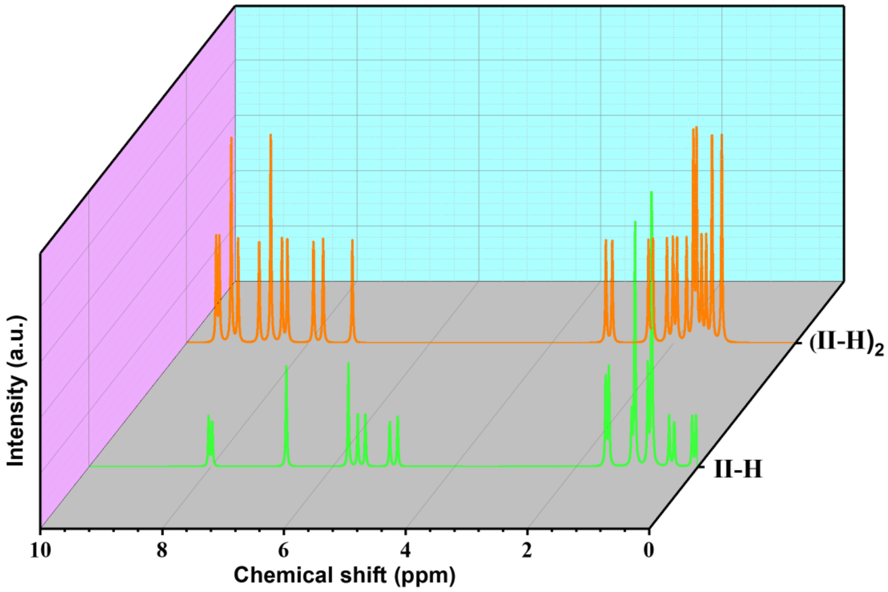

(d)

Figure S3. Simulated Nuclear Magnetic Resonance Hydrogen (HNMR) Spectrum of (a) and (b) complexes Ⅰ and refer to the intermediates of Ⅰ$^-$, Ⅰ-H and (Ⅰ-H)$^-$; (c) and (d) complexes Ⅱ and refer to the intermediates of Ⅱ$^-$, Ⅱ-H and (Ⅱ-H)$_2$.

Note: (1) Chemical shifts of ^{1}H were corrected using tetramethylsilane (TMS) obtained under PBE0/def2-SVP conditions as reference values for complexes Ⅰ and Ⅱ and their respective reaction intermediates; (2) red (Ⅰ) and (Ⅱ) refer to the reference experimental spectrum[1].

References:

[1] X. Guo, N. Wang, X. Li, Z. Zhang, J. Zhao, W. Ren, S. Ding, G. Xu, J. Li, U.P. Apfel, W. Zhang, R. Cao, Homolytic versus Heterolytic Hydrogen Evolution Reaction Steered by a Steric Effect, Angewandte Chemie International Edition, 59 (2020) 8941-8946.
